# A 5-year longitudinal study of survival rate and periodontal parameter changes at sites of dilacerated maxillary central incisors

**DOI:** 10.1186/2196-1042-15-3

**Published:** 2014-01-06

**Authors:** Giampietro Farronato, Lucia Giannini, Guido Galbiati, Cinzia Maspero

**Affiliations:** Department of Orthodontics, IRCCS Cà Granda – Ospedale Maggiore Policlinico, University of Milan, Via Commenda 10, 20100 Milano, Italy

**Keywords:** Dilaceration, Clinical periodontal parameter, Disinclusion

## Abstract

**Background:**

Although dental dilaceration disinclusion is an accepted treatment modality, few studies have evaluated the prognosis for dilacerated maxillary incisors and changes in clinical periodontal parameters still need to be demonstrated. The objectives of this study were to evaluate the prognosis and changes in clinical attachment level (CAL), probing depth (PD), and soft tissue recession (REC) for disincluded dilacerated maxillary incisors.

**Methods:**

Ten impacted dilacerated teeth were scheduled for disinclusion. Five of them were disincluded with the apically repositioned flap technique and the other five with the closed eruption technique and brought into alignment with light orthodontic forces.

**Results:**

All the dilacerated teeth were disincluded as planned. CAL, PD, and REC were the same as natural teeth. Among the two surgical techniques, no statistically significant differences have been found.

**Conclusion:**

The choice to disinclude dilacerated central maxillary incisors reached the goals planned. Periodontal parameters obtained in a short- and long-term follow-up allow to affirm that the disinclusion of dilacerated teeth has a good survival rate.

## Background

Dilaceration is a dental deformity characterized by an angulation between crown and root causing non-eruption of the tooth [1, 2]. This deformity is due to a disturbance between mineralized and mineralized portions of the developing tooth germ [3].

Surgical extraction used to be the first choice in treating the severely dilacerated teeth [1]. In addition, many reports have been described to move dilacerated teeth into alignment. The therapeutic approach to the dilacerated maxillary central incisors has to be carefully planned and needs the cooperation of several specialities to attain the final objectives. The chances of failure could be due to ankylosis, external root resorption and root exposure after orthodontic traction [4, 5]. Even the successful cases probably have an unesthetic gingiva of the exposed incisor and need to have periodontal surgery [6].

Experimental and clinical studies confirmed that disinclusion of dilacerated teeth is a secure and useful treatment modality and that dilacerated teeth have the capacity for functional adaptation and preservation of the alveolar ridges and periodontal tissues. The follow-up periods and the types of dilacerated teeth are different in each study, and reported survival rates are variable. Favorable healing of the periodontal ligament (PDL) depends on how many viable cells are preserved on the roots. These cells are easily injured under stressful conditions such as variable pH, osmotic pressure, and dehydration.

There are few studies evaluating the prognosis for dilacerated maxillary incisors and changes in clinical periodontal parameters after disinclusion, including clinical attachment level (CAL) and probing depth (PD), need to be demonstrated.

The aims of this retrospective study were to evaluate the prognosis for dilacerated maxillary incisors and to evaluate changes in clinical periodontal parameters, including CAL, PD, and soft tissue recession (REC).

## Methods

### Study population

The sample included 10 patients (6 females, 4 males), with ages ranging from 25 to 35 years, which underwent disinclusion of dilacerated central incisors.

The inclusion criteria were

-  Unremarkable medical history
-  Caucasian ethnicity
-  Availability for scheduled appointments
-  Pre-treatment and post-treatment X-ray with excellent contrast
-  Pre-treatment and post-treatment study casts
-  Availability of a signed and written informed consent statement

The exclusion criteria were

-  Congenital and dental anomalies
-  Systemic or local condition that might have jeopardized the results
-  Failure to attend more than two appointments

Ten maxillary incisors were surgically exposed and brought into proper position between 2001 and 2007. The patients enrolled in the retrospective study were divided into two groups according to the surgical technique chosen by the surgeon.

In five patients, the maxillary incisors were exposed through a closed eruption technique (group A) and in the other five through an apically positioned flap technique (group B).

In the patients where the closed eruption technique was performed, a flap was created to expose the lingual surface of the crown of the dilacerated tooth, a lingual button was attached with a 0.010-inch ligature wire on it. The flap was reclosed and sutured, leaving a tied ligature wire with a hook end protruding through the mucosa. The apically repositioned flap technique involved a split-thickness pedicle flap reflected from the edentulous area extended vertically into the vestibule. The bone covering the dental crown was removed with a surgical round bur. This allowed the palatal surface of the right central incisor to be exposed. The flap was then repositioned apically and sutured to the periosteum adhering to the surrounding gingiva and leaving two thirds of the crown of the tooth uncovered.

A light force was applied by an elastomeric chain. As the dilacerated tooth moved downward, the ligature wire was cut shorter to maintain the effective elastomeric chain. The same procedure of elastic traction continued until the impacted tooth became exposed to the oral environment. The attached button and bracket were then removed and a standard incisor bracket was bonded so that the tooth could be properly positioned. The final alignment was completed and an ideal overbite and overjet was established.

The post-surgical follow-up and maintenance periods were carried out to achieve the best possible plaque control.

### Clinical measurements

A clinical examination was performed at the time of bracket removal both 1 and 5 years after the therapy. The presence of plaque was assessed at every examination during the first year. PD, CAL, and REC were recorded at T0 (beginning), T1 (1 year - postoperative) and T2 (5 years - postoperatively). All measurements were performed by the same examiner at four sites of the tooth (mesial, distal, buccal, and palatal) and recorded to the nearest millimeter (Tables 1, 2, 3, and 4).

**Table 1:** **Clinical and radiographic data in the four sites**

Clinical assessment	Mesial		Buccal		Distal		Palatal		Overall	
	Mean	SD	Mean	SD	Mean	SD	Mean	SD	Mean	SD
**PD**										
T0	1.93	*0.62*	1.37	0.71	1.83	0.69	1.62	0.67	1.6875	0.247975
T1	1.92	*0.63*	1.36	0.69	1.82	0.67	1.59	0.61	1.6725	0.249983
T2	1.92	*0.71*	1.35	0.68	1.82	0.57	1.58	0.63	1.6675	0.255261
**CAL**										
T0	2.18	*0.71*	1.57	0.63	2.1	0.69	1.98	0.8	1.9575	0.271093
T1	2.19	*0.73*	1.59	0.62	2.12	0.67	1.99	0.89	1.9725	0.268126
T2	2.21	*0.74*	1.6	0.6	2.13	0.68	1.99	0.87	1.9825	0.270724
**REC**										
T0	0.25	*0.58*	0.2	0.4	0.27	0.59	0.36	0.61	0.27	0.066833
T1	0.27	*0.57*	0.23	0.41	0.3	0.57	0.4	0.63	0.3	0.072572
T2	0.29	*0.58*	0.25	0.4	0.31	0.57	0.41	0.62	0.315	0.068069

Patients treated with closed eruption technique. PD, probing depth; CAL, clinical attachment level; REC, soft tissue recession.

**Table 2:** **Clinical and radiographic data in the four sites**

Clinical assessment	Mesial		Buccal		Distal		Palatal		Overall	
	Mean	SD	Mean	SD	Mean	SD	Mean	SD	Mean	SD
T0	1.91	0.61	1.35	0.72	1.85	0.68	1.63	0.67	1.685	0.253706
T1	1.93	0.63	1.36	0.69	1.84	0.67	1.6	0.62	1.6825	0.256174
T2	1.92	0.69	1.35	0.68	1.83	0.62	1.59	0.64	1.6725	0.256174
**CAL**										
T0	2.19	0.72	1.57	0.64	2.09	0.7	1.97	0.82	1.955	0.271968
T1	2.19	0.73	1.6	0.62	2.11	0.67	1.99	0.89	1.9725	0.261582
T2	2.2	0.73	1.6	0.61	2.13	0.69	1.99	0.88	1.98	0.267955
**REC**										
T0	0.27	0.59	0.21	0.41	0.24	0.61	0.35	0.61	0.2675	0.060208
T1	0.27	0.57	0.23	0.41	0.28	0.57	0.37	0.67	0.2875	0.05909
T2	0.29	0.59	0.26	0.43	0.3	0.59	0.4	0.64	0.3125	0.060759

Patients treated with apically positioned flap technique.

**Table 3:** **Clinical and radiographic data overall and changes from T1 to T2**

Clinical assessment	Mean	SD	T1 to T2
**PD**			
T0	1.6875	0.247975	
T1	1.6725	0.249983	
T2	1.6675	0.255261	−0.005
**CAL**			
T0	1.9575	0.271093	
T1	1.9725	0.268126	
T2	1.9825	0.270724	0.01
**REC**			
T0	0.27	0.066833	
T1	0.3	0.072572	
T2	0.315	0.068069	0.015

Patients treated with closed eruption technique. Statistically significant differences are presented in italics.

**Table 4:** **Clinical and radiographic data overall and changes from T1 to T2**

Clinical assessment	Mean	SD	T1 to T2
**PD**			
T0	1.685	0.253706	
T1	1.6825	0.256174	
T2	1.6725	0.256174	−0.01
**CAL**			
T0	1.955	0.271968	
T1	1.9725	0.261582	
T2	1.98	0.267955	0.0075
**REC**			
T0	0.2675	0.060208	
T1	0.2875	0.05909	
T2	0.3125	0.060759	0.025

Patients treated with apically positioned flap technique. Statistically significant differences are presented in italics.

For all the measures, a calibrated periodontal probe has been used.

Each measurement has been performed two times by the same operator and the medium value has been considered.

If patients showed evidence of non-compliance with oral hygiene, measures and a follow-up appointment was made to ensure adequate plaque control. Professional supragingival tooth cleaning was performed when needed. Neither probing nor subgingival instrumentation was performed during the first year after surgery.

### Statistical analysis

The available data from all the examination were statistically analyzed to evaluate the prognosis and survival rate of the dilacerated teeth. Means and standard deviation for CAL, PD, and REC were calculated during the 5 years of observation. A paired *t* test was used to compare T0 with T1 and T1 with T2 values.

The first comparison aimed to demonstrate if there was a difference from the time before the surgical intervention and after it; the second one aimed to demonstrate if the results were stable during the follow-up period. We did not compare the two surgical techniques statistically because the aim of the study was to evaluate the stability in time (difference between T1 and T2) of the two techniques and not the pure result obtained.

Also, the results obtained with the closed eruption techniques were compared with the ones obtained with those of the apically positioned flap technique.

An error of <0.05 was accepted as statistically significant. The statistical tests were performed using the SPSS program.

The method error was determined using Dahlberg's formula ME = √∑*d*2/2*n*, where *n* is the number of subjects and *d* is the difference between the two measures. The method error did not exceed 0.1 mm for the linear measurements and 0.2° for angular measurements.

## Results

### Clinical findings

All the dilacerated teeth were disincluded as planned, and all surgical sites healed with no evidence of postoperative infection. All the subjects were happy with the esthetic results. The appearance of the gingiva and alveolar mucosa did not differ from that of the adjacent teeth. The normal structure of the gingiva was formed within the first year after disinclusion and remained constant during the follow-up period.

### Survival rate

The follow-up period was 5 years. All teeth are still under the maintenance program.

### Clinical measurements

PD, CAL, and REC values are shown in Tables 1, 2, 3, and 4.

Among the two surgical techniques, no statistically significant differences have been found.

Analyzing data obtained, it can be underlined that from T0 to T1, there is a statistically significant difference among the examination, while from T1 to T2, there are none. So, the results obtained just after the disinclusion can improve during the first year and then remain stable during time.

All the parameters analyzed showed a significant improvement from the period after the disinclusion and 1-year follow up and then they remain constant during the time.

These considerations can be observed both in the overall measurements and in every site (buccal/palatal/mesial/distal).

## Discussion

In literature, there are currently contrasting opinions on the therapeutic choice for cases of dilacerated teeth.

The most commonly mentioned therapeutic solution in literature is surgical extraction and substitution with Maryland-bridge or prosthetic implants [7, 8] because of the technical difficulty involved in exposition and orthodontic alignment and the uncertain prognosis of such malformed teeth.

It is therefore difficult to establish the prognosis of impacted and dilacerated teeth when disincluded [9, 10]. This difficulty is enhanced when the tooth involved is a central maxillary incisor. Prognosis depends on both the seriousness and position of the dilacerations, as well as the formation of the root.

The follow-up results in the present study indicated a favorable prognosis for the dilacerated disincluded maxillary incisors if the treatment is performed with a meticulous surgical technique and appropriate post-operative control, both with apically positioned flap and closed eruption technique.

Disinclusion of impacted or dilacerated tooth can be facilitated by rapid palatal expansion to increase arch length [11–15].

The two different surgical approaches, if planned and performed in a good manner, do not influence the results obtained both in a short-term than in a long-term follow-up.

Healing in soft tissues was good and rapid at the end of therapy, and PDL space around the disincluded teeth could be seen on radiographs.

The results of the current study suggest that a normal gingival tissue, with its usual consistency, color, and stippled appearance, is established parallel with bone healing, regardless of the preoperative condition of gingival and alveolar mucosa.

Favorable healing of PDL depends on how many viable cells are preserved on the root; our findings suggested that clinically, satisfactory healing also takes place in the dilacerated disincluded teeth. From a periodontal point of view, CAL and PD values were observed at approximal sites.

Compared to the immediate post-treatment, all mesial, buccal, distal and palatal measurements and the mean CAL, PD, and REC values increased slightly during the follow-up period (1 year) and remain stable during the subsequent period, as shown by the measurement taken after 5 years.

These increases might have been due to continued maturation, the aging process, or organization of periodontal tissues after the teeth started to function.

All clinical measurements were as stable at the 5-year observation as a natural tooth.

Within the limits of this study, it was confirmed that disincluded dilacerated teeth simulate natural teeth, maintaining healthy periodontal support. With such a procedure, extractions can be avoided.

## Conclusion

Determining prognosis and designing a treatment plan for a dilacerated impacted tooth, especially in an adult patient, is often a difficult task. The easiest chosen therapy is surgical extraction, but the disinclusion offers better esthetic and functional results.

In this study, it was confirmed that it is possible to disinclude dilacerated teeth with optimal periodontal results.

The factors that influence the positive outcomes are

1. The surgical technique freeing the fibrous tissue, blocking eruption.
2. Use of light and constant orthodontic forces (30 to 40 g).
3. The favorable crown-root angle allowing the crown to be aligned without excessive dislocation of the root.

Although this final aspect of the tooth can only be evaluated with precision during treatment, the orthodontic-surgical option is a possible technique guaranteeing sustainable results stable during time.
